# Dissemination, implementation and impact of the ESHRE evidence-based guidelines

**DOI:** 10.1093/hropen/hoz011

**Published:** 2019-06-06

**Authors:** S Gameiro, M Sousa-Leite, N Vermeulen

**Affiliations:** 1 Cardiff Fertility Studies Group, School of Psychology, Cardiff University, Cardiff, UK; 2 School of Psychology, Minho University Campus de Gualtar, Braga, Portugal; 3 European Society for Human Reproduction and Embryology, Grimbergen, Brussels, Belgium

**Keywords:** endometriosis, premature ovarian failure, recurrent miscarriage, psychology, counselling, assisted reproduction, infertility, ESHRE

## Abstract

**STUDY QUESTION:**

What are the perceptions of ESHRE members about the dissemination, implementation and impact of the first four ESHRE evidence-based guidelines to be published?

**SUMMARY ANSWER:**

Around 30% of ESHRE members know and use the ESHRE evidence-based guidelines in their routine practice and this is perceived to result in better treatment, better screening/evaluation/diagnosis and better psychosocial and patient-centred care, with on average three in each four members who make changes perceiving that their patients benefit from it.

**WHAT IS KNOWN ALREADY:**

ESHRE has been developing and disseminating evidence-based guidelines, aiming to improve the quality of fertility care across Europe. However, evidence has shown that guidelines dissemination is not enough to change practice at clinics, with implementation strategies that address local barriers to implementation being recommended.

**STUDY DESIGN, SIZE, DURATION:**

A cross-sectional study based on an online survey was sent by email to all ESHRE members (*n* = 7664) and advertised on ESHRE social media (20 February–3 April 2018). The survey was carried out to evaluate their perceptions about the dissemination, implementation and impact of the Management of Endometriosis (ENDO), Routine Psychosocial Care (RPC), Premature Ovarian Insufficiency (POI) and Recurrent Pregnancy Loss (RPL) ESHRE guidelines.

**PARTICIPANTS/MATERIALS, SETTING, METHODS:**

The survey was advertised via the ESHRE website, social media and email to all ESHRE members. It assessed the dissemination (knowledge the guidelines were published, downloaded), implementation (using guidelines in daily practice, changed practice) and impact (perceived patient benefit, referred patients to the guidelines) of the guidelines, as well as their perceived implementability. Open questions assessed perceived changes in practice, barriers to and desired support for implementation.

**MAIN RESULTS AND THE ROLE OF CHANCE:**

The final sample consisted of 658 participants (not possible to calculate response rate), with the majority being embryologists, biologists or geneticists (*n* = 268, 40.7%), followed by clinicians (*n* = 260, 39.5%), scientists (*n* = 48, 7.3%), nurses or midwives (*n* = 30, 4.6%), psychologists, counsellors or social workers (*n* = 28, 4.3%) and others (e.g. medical student, lab manager, marketing, ethicist; *n* = 24, 3.6%). The majority knew that ESHRE published the guidelines (82.1% ENDO, 54.6% RPC, 56.6% POI, 59.4% RPL). From these, the majority downloaded it (65.9% ENDO, 52.4% RPC, 54.2% POI, 56.8% RPL), around one-third used it in their routine practice (41.7% ENDO, 29.5% RPC, 33.7% POI) and around one quarter made changes to their practice (30.7% ENDO, 18.9% RPC, 21.5% POI). Overall, <20% of members think that patients benefited from the guideline (19.4% ENDO, 16.3% RPC, 16.1% POI) and very few referred them to it (ENDO 8.9%, 12.8% RPC, 16.1% POI). However, on average every three in every four people who made changes to practice perceived that their patients benefited from it (ENDO 62%, RPC 80%, POI 75%). The main reported changes in practice were better treatment, better screening/evaluation/diagnosis and better psychosocial and patient-centred care. Main perceived barriers to implementation were lack of translation to other languages, guidelines being long and difficult to understand and lack of supporting evidence. Financial constraints and lack of staff expertise were also reported. Participants desired clear support for implementation in the form of step-by-step instructions, more training and support materials for staff and patients and translation to other languages. Results for the clinicians only showed that, despite less knowledge about the RPC guideline, they were more likely to download all the guidelines, to follow them, make changes in their daily practice and refer them to their patients.

**LIMITATIONS, REASONS FOR CAUTION:**

Respondents were ESHRE members and these are not representative of all European reproductive health professionals. The response rate could not be calculated as ESHRE social media reaches more than just the members. The guidelines are mainly written for clinicians and in this sample the clinicians were under-represented. In addition, missing values increased as participants progressed through each guideline’s questions, with the open-ended questions being answered by only 74–97 participants. The survey assessed perceptions instead of actual practice. Overall, the results may convey a too optimistic picture of the impact of the guidelines.

**WIDER IMPLICATIONS OF THE FINDINGS:**

ESHRE’s policy of investing in implementation and dissemination is important but insufficient to ensure the guidelines are implemented at clinics across Europe. ESHRE can address perceived barriers that are directly related to the guidelines, in particular lack of translation, as well as provide further support for implementation. This support should be clear and concise, focusing on how to implement the guidelines rather than on what to do.

**STUDY FUNDING/COMPETING INTEREST(S):**

None.

**WHAT DOES THIS MEAN FOR PATIENTS?**

Evidence-based guidelines are a set of recommendations about how staff in fertility clinics should provide care to their patients, which are based on the most high quality and up-to-date scientific research. ESHRE has been developing evidence-based guidelines in an effort to improve the quality of fertility care across Europe. However, publishing and advertising guidelines is not enough to ensure that fertility staff will actually follow the recommendations when attending to their patients.

We did an online survey to ask ESHRE members if they know about and follow the ESHRE guidelines, and if they think their patients benefit from this. We also asked them about how following the guidelines changed their practice, what makes it difficult for them to use the guidelines and what support they think could be beneficial.

Results from the survey show that the majority of ESHRE members know the guidelines are available, but only ~30% follow them. Those who follow the guidelines think that they are providing better treatment, better diagnosis and better psychosocial and patient-centred care to their patients. On average, three in every four members who made changes to their daily practice perceived that their patients benefited from it. However, the guidelines are long and difficult to understand and are usually not translated to other languages (beyond English). If ESHRE is able to address these issues, in particular translation of the guidelines, fertility staff may feel more empowered to follow the guidelines, and this can mean better care for their patients.

## Introduction

Between 2013 and 2017 ESHRE published four systematically developed and evidence-based clinical practice guidelines [Management of Endometriosis (ENDO; Dunselman *et al*., 2014), Routine Psychosocial Care (RPC; Gameiro *et al*., 2015), Premature Ovarian Insufficiency (POI; Webber *et al*., 2016) and Recurrent Pregnancy Loss (RPL) (Atik *et al*., 2018)] with the aim of supporting care providers and patients making every-day decisions about appropriate and effective health care (Vermeulen *et al*., 2017). Since then ESHRE has endorsed one more evidence-based guideline and has three under development. The goal of the present study is to evaluate the dissemination, implementation and impact of the first four published ESHRE guidelines.

ESHRE’s motivation to develop clinical practice guidelines is ‘to improve the quality of health care delivery within the European field of human reproduction and embryology’ (Vermeulen *et al*., 2017, p. 5). With this aim in view, the development of all ESHRE guidelines is based on a 12-step systematic process intended to maximize their implementability at clinics (Kashyap *et al*., 2011). The initial steps of this process include an exhaustive review and comprehensive synthesis of the best available relevant evidence and its quality assessment, in order to ensure that the recommendations developed are valid (reflect the strength of evidence available) and measurable (identify outcomes to measure the effect of implementation). The subsequent steps in the process aim at the adequate translation of the evidence into a set of best practice recommendations designed to ensure, among other characteristics, their executability (say exactly what to do), decidability (say precisely under what conditions) and flexibility (allow for interpretation and alternatives in execution). This last two steps of a stakeholder consultation focusing on ensuring that the clinical content of the recommendations and their applicability are adequate, followed by the final approval of the resulting document by the ESHRE Executive Committee (Vermeulen *et al*., 2017).

ESHRE has also been making a very significant investment in the dissemination of the guidelines, which includes making them available online in various professional and patient-friendly formats (e.g. via the ESHRE website, ESHRE journals, a pocketsize version), advertising and distributing them widely (e.g. social media, ESHRE meetings, via national societies), developing apps and online training courses and working collaboratively with multiple national societies to promote their endorsement and translation into different languages. As an example, the RPC guideline (https://www.eshre.eu/Guidelines-and-Legal/Guidelines/Psychosocial-care-guideline.aspx) has been published in five formats (full document, summary document, patient versions, pocketsize version and a paper in *Human Reproduction*) and is currently translated into Dutch, Greek and Spanish. ESHRE has also made available additional educational materials and produced podcasts focusing on different aspects of these guidelines and is currently developing an e-campus course with the aim of supporting fertility staff in their day-to-day implementation.

The implementation of trustworthy guidelines should reduce inappropriate practice variation (Institute of Medicine, 2011), but so far ESHRE has put little emphasis on ensuring that its guidelines are being implemented across Europe. It is well established that publishing and disseminating guidelines is not enough for health care providers to change their daily practice towards guidelines adherence. However, it is also clear that no single implementation strategy will efficiently ensure implementation at a European level. Indeed, implementation is known to vary nationally and locally depending on existing barriers related to the guidelines themselves (e.g. is it translated to the country’s language), the health care providers (e.g. staff attitudes and expertise), the social and clinical setting (e.g. clinic’s characteristics) and the system (e.g. legal, financial constrains; Shiffman *et al*., 2005). The consequence is that efficient implementation strategies need to target the specific barriers affecting local practice (Baker *et al*., 2010). It is based on this argument that ESHRE has taken responsibility for the implementability of its guidelines (i.e. ensuring that they are implementable), but not directly for their implementation into local practice.

The current study reports on an online survey designed to provide preliminary evidence of the implementability and implementation of the first four published ESHRE guidelines (ENDO, RPC, POI and RPL). More specifically, the survey assesses dissemination (knowledge that the guidelines exist and access to them), implementation (using guidelines and making changes to practice according to guideline recommendations) and impact (referral of patients to the patient-friendly version of guidelines, perceived patient benefit). It further investigates the perceived implementability of the guidelines (across multiple dimensions), barriers to implementation and perceived beneficial support for their implementation. The results will indicate the extent to which the ESHRE guidelines are being used across Europe (and beyond) to promote evidence-based clinical decision-making and improve fertility healthcare quality and safety.

## Materials and Methods

### Study design

A cross-sectional study was conducted using an online survey, which focused on the first four evidence-based guidelines published by ESHRE (ENDO, RPC, POI and RPL).

### The survey

The survey was organized in two sections: the first focused on the participants’ background and the second on the guidelines. For each guideline, participants were presented with questions focusing on the following: dissemination, implementation and impact of the guidelines; their implementability; changes made in practice as a result of implementing the guidelines; and perceived barriers and desired support for implementing the guidelines. These questions were presented sequentially in the following order: ENDO, RPC, POI and RPL. Because the RPL guidelines had just been published, a few questions were omitted or adapted, and this is described below, where we present the materials in more detail.

#### Background variables

Participants were asked about their age (in years), gender (male; female; other), country of work (collapsed into continents) and professional background (clinician; embryologist, biologist or geneticist; scientist; nurse or midwife; psychologist, counsellor or social worker; and other).

#### Dissemination, implementation and impact of the guidelines

Participants were asked the following:

- two dissemination questions i.e. if they knew that the guideline was published (no; yes) and if they had downloaded it (no; yes)
- two implementation questions i.e. if they were using these guidelines in their routine practice (no; yes) and if they or the clinic had made changes in their routine practice as a result of applying them (no; yes)
- two impact questions i.e. to what degree they thought patients had benefited from the changes implemented (1, not at all; 2, a little bit; 3, somewhat; 4, quite a bit; 5, a tremendous amount, also recoded into no [not at all]; yes [from a little bit to a tremendous amount]) and if they had referred their patients to the patient version of the guidelines (no; yes).

The implementation and impact questions were omitted for the RPL guideline and only one implementation question was asked, if participants intended to make changes at their clinic based on the guideline (no; yes).

#### Implementability of the guidelines

Patients were asked to rate the ENDO, RPC and POI guidelines according to the six dimensions of the Guideline Implementability Appraisal tool (GLIA; Kashyap *et al*., 2011), namely executability, decidability, validity, flexibility, effect on process of care and measurability, on a 5-point Likert scale (from 1, very poor to 5, excellent). Patients were also asked to provide an overall rating of the four guidelines using the same response scale.

#### Changes in practice, barriers to implementation and desired support for implementation

Participants were presented with a series of open-ended questions, where they were asked to provide up to three important examples of how applying the guidelines changed their practice, perceived barriers for applying the guidelines (for each of the following headings: the guidelines themselves, the clinical setting, the staff and patients) and types of desired support to implement the guidelines (for each of the headings listed above). None of these questions were presented for the RPL guideline. Finally, participants were asked about their intentions (yes; maybe; no; do not know) to use different types of implementation support provided by ESHRE, namely e-learning course, campus course, guidelines App, printed pocket guidelines and a step-by-step guide.

### Procedure

The survey was posted online from 20 February to 3 April 2018. Multiple recruitment strategies were used. The study was advertised via two emails to the ESHRE membership (*n* = 6764) and via the ESHRE social media. Participants were offered the opportunity to win a free registration for the ESHRE Annual Meeting in Barcelona or an ESHRE Campus Course.

### Statistical analysis

Descriptive statistics were performed to analyse participants’ background, the dissemination, implementation and impact of the guidelines, their perceived implementability and intentions to use support provided by ESHRE.

To investigate the factors associated with the use of the guidelines (dissemination, implementation and impact) multiple logistic regressions were conducted. The following factors were considered: age, gender (0, male; 1, female or other), professional background (0, non-clinician; 1, clinician), continent (0, non-European; 1, European) and overall quality rating of the guideline. For each guideline these factors were regressed on the two indicators of dissemination, implementation and impact (total of six regressions per guideline). For the RPL guideline only the two indicators of dissemination and the future orientated implementation question were considered (total of three regressions).

To investigate perceived changes in practice, barriers to and desired support for implementation of the ENDO, RPC and POI guidelines, content analysis on the participants’ open-ended responses was conducted. Each participant could contribute up to 11 replies to the group data, 3 to changes made at clinic, 4 to perceived barriers and 4 to desired support. We first checked if replies had text that could be coded (the percentage of non-codable answers is presented in results). Inductive coding was then applied for each question, meaning that participants’ answers were grouped into meaningful categories, on the assumption that answers assigned to the same category shared the same meaning (Cavanagh, 1997). An answer could contain text referring to different barriers or support and therefore these could be assigned to more than one category (maximum of two was observed). This process was carried out independently by S.G. and M.S.L. who then came together to review their coding. Disagreement about the coding of each answer or the labels created for the emergent categories was resolved by discussion until consensus was achieved. Although the answers for perceived barriers and desired support were given under specific headings, they were sometimes placed under a different one. For instance, the following reply was presented as a patient barrier: ‘Lack of patient knowledge due to language barrier (not all patients can understand English)’ but was then placed under guidelines barriers, as it is not the patient that should adapt to the guidelines but the guidelines to the patient population. In the third step, N.V. was asked to review the coding and suggestions for changes were discussed by the three authors until consensus was achieved. Finally, descriptive statistics were used to provide the frequency of the emergent categories for each guideline.

Quantitative data analyses were performed with IBM© SPSS© Statistics Version 25 (IBM-SPSS, Chicago, IL, USA). No software was used to perform qualitative analyses.

## Results

### Participants

A total of 723 people accessed the survey. Of these, 65 participants were excluded because they only filled the background questions. The final sample consisted of 658 participants.

Participants were on average 42.91 years old (*SD* = 11.11, range 20–78). The majority were women (*n* = 396, 60.2%), with the remaining being men (*n* = 253, 38.4%) and one participant stating other (0.2%). The participants were from 95 different countries, and the majority were based in Europe (*n* = 429, 65.2%), followed by Asia (*n* = 140, 21.3%), South America (*n* = 31, 4.1%), Africa (*n* = 30, 4.6%), North America (*n* = 18, 2.7%) and Oceania (*n* = 10, 1.5%). The participants had different professional backgrounds, with the majority being embryologists, biologists or geneticists (*n* = 268, 40.7%), followed by clinicians (*n* = 260, 39.5%), scientists (*n* = 48, 7.3%), nurses or midwives (*n* = 30, 4.6%), psychologists, counsellors or social workers (*n* = 28, 4.3%) and others (e.g. medical student, lab manager, marketing, ethicist; *n* = 24, 3.6%). We contrasted these descriptive statistics with the total ESHRE membership (data not reported). Overall, the sample seems representative of ESHRE’s membership; however, embryologists, biologists or geneticists seem to be overrepresented (40.7% versus 30.8% in ESHRE membership).

### Dissemination, implementation and impact of the guidelines

Participants had to answer multiple questions for each guideline and address each of the four guidelines in a sequential order (ENDO, RPC, POI and RPL). The result was that the survey was quite time-consuming and participants tended to stop answering it at the end of each guideline, with missing answers increasing sequentially (ENDO: 0, 0%; RPC: 307, 42.5%; POI: 361, 49.9%; and RPL: 388, 53.7%).

Table I presents the results regarding the dissemination, implementation and impact of the four published ESHRE guidelines (see Supplementary Table SX for results for clinicians only). The majority of the participants (≥54.6%) knew that ESHRE had published the guidelines. From these, the majority (≥52.4%) had downloaded the four guidelines, around one-third used the first three in their daily practice and around one-fourth reported that they made or intend to make changes to their practice. For the ENDO, RPC and POI guidelines, only a minority perceived that their patients benefited from the guidelines and referred them to the guidelines.

### Factors associated with use of the guidelines

**Figure 1:**
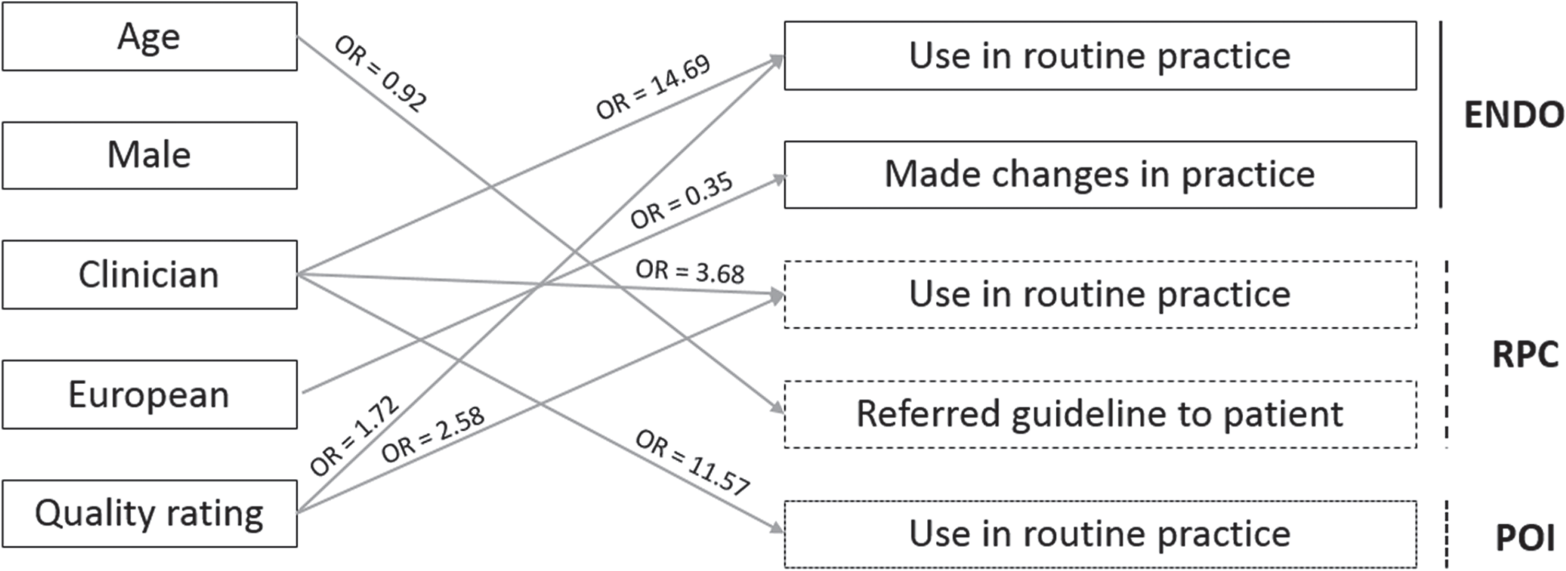
**Schematic representation of the statistically significant associations (*P* < 0.05) found between the predictors of the guidelines’ use and the dissemination, implementation and impact indicators.** OR, odd ratios. Guidelines: ENDO, Endometriosis; RPC, Routine Psychosocial Care; POI, Premature Ovarian Insufficient. See text for full results reporting.

Figure 1 presents a schematic representation of the significant associations found between the factors that were considered to be associated with use of the guidelines and the dissemination, implementation and impact indicators.

**Table I:** Dissemination, implementation and impact of the four published ESHRE guidelines.

		**ENDO**	**RPC**	**POI**	**RPL**
Know was published	q/n^a^	540/658	227/416	205/362	199/335
	% [95% CI]	82.07 [78.95–84.81]	54.57 [49.76–59.29]	56.63 [51.48–61.64]	59.40 [54.07–64.53]
Downloaded	q/n^b^	356/540	119/227	111/205	113/199
	% [95% CI]	65.93 [61.83–69.80]	52.42 [45.94–58.83]	54.15 [47.31–60.83]	56.78 [49.84–63.47]
Use in daily practice	q/n^b^	225/540	67/227	69/205	–
	% [95% CI]	41.67 [37.58–45.87]	29.52 [23.96–35.75]	33.66 [27.54–40.38]	–
Changes in practice	q/n^b^	166/540	43/227	44/205	63/199
	% [95% CI]	30.74 [27.00–34.76]	18.94 [14.38–24.54]	21.46 [16.40–27.58]	31.66 [25.60–38.42]
Perceived patient benefit	q/n^b^	105/540	37/227	33/205	–
	% [95% CI]	19.44 [16.33–22.99]	16.30 [12.06–21.66]	16.10 [11.70–21.74]	–
	M	4.12	4.30	4.39	–
	SD	0.91	0.74	0.86	–
Referred to patients	q/n^b^	48/540	29/227	33/205	–
	% [95% CI]	8.89 [6.77–11.59]	12.78 [9.04–17.75]	16.10 [11.70–21.74]	–

*N* = 658 participants;

q, number of participants replying yes to question.

^a^n, number of valid answers (i.e. not missing); ENDO, 658; RPC, 416; POI, 362; RPL, 335.

^b^n, number of participants who reported knowing the guidelines were published; ENDO, 540; RPC, 227; POI, 205; RPL, 199.

ENDO, Endometriosis; RPC, Routine Psychosocial Care; POI, Premature Ovarian Insufficiency; RPL, Recurrent Pregnancy Loss.

**Figure 2:**
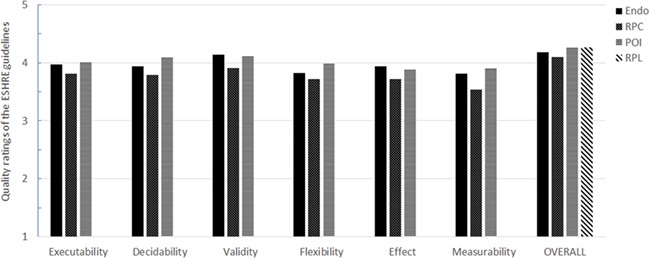
**Quality ratings of the guidelines for the Guideline Implementability Appraisal tool implementability dimensions and overall.** RPL, Recurrent Pregnancy Loss.

**Table II:** Reported perceived changes in practice due to implementing the guidelines.

	**ENDO** (*n* = 233), *n* (%)	**RPC** (*n* = 68), *n* (%)	**POI** (*n* = 50), *n* (%)
Increased awareness/knowledge	6 (2.58)	12 (17.65)	6 (12.00)
Better screening/evaluation/diagnosis	21 (9.01)	8 (11.76)	11 (22.00)
Better general treatment	71 (30.47)		16 (32.00)
Better medical treatment of pain	20 (8.58)		
Better surgical treatment	54 (23.18)		
Better psychosocial support/counselling	6 (2.58)	16 (23.53)	4 (8.00)
Better patient-centred care	12 (5.15)	7 (10.29)	4 (8.00)
Better education/information provision	19 (8.15)	2 (2.94)	5 (10.00)
Better communication		4 (5.88)	
Endometriosis advocacy	2 (0.86)		
More interdisciplinary/team work		6 (8.82)	
Clinical auditing	1 (0.43)	1 (1.47)	
Reduced costs	2 (0.86)		
New infrastructures		1 (1.47)	
Non-codable	19 (8.15)	11 (16.18)	4 (8.00)

See Supplementary Tables SI/SIV/SVII for the full list of the participants’ answers and the respective category.

**Table III:** Reported barriers to implementing the guidelines.

	**ENDO** (*n* = 223), *n* (%)	**RPC** (*n* = 82), *n* (%)	**POI** (*n* = 63), *n* (%)
**Guidelines**			
Unclear/difficult to understand	7 (3.14)	4 (4.88)	4 (6.35)
Too long	18 (8.07)	3 (3.66)	3 (4.76)
Lack of evidence	10 (4.48)	5 (6.10)	3 (4.76)
Language/translation	10 (4.48)	10 (12.20)	8 (12.70)
Topic not relevant/inappropriate/not priority	5 (2.24)	3 (3.66)	1 (1.59)
Lack of specific information	6 (2.69)		
Lack of patient-friendly version/materials	5 (2.24)		1 (1.59)
No dissemination/awareness	1 (0.45)		3 (4.76)
Lack of power	5 (2.24)		
**Clinical setting and system**			
Costs/financial constrains	16 (7.17)	6 (7.32)	4 (6.35)
Lack of infrastructure/equipment	9 (4.04)	1 (1.22)	
Lack of trained/specialised staff	4 (1.79)	1 (1.22)	
Culture/norms	3 (1.35)	5 (6.10)	
No champion/responsible person		1 (1.22)	
Not relevant to clinic patient population		1 (1.22)	
**Staff**			
Lack of knowledge/expertise	19 (8.52)	5 (6.10)	4 (6.35)
Lack of time	8 (3.59)	7 (8.54)	2 (3.17)
Lack of power		1 (1.22)	
Lack of interest/motivation	2 (0.90)	3 (3.66)	
Not personally applicable/relevant			2 (3.17)
Culture/norms	3 (1.35)		
**Patient**			
Culture/norms	10 (4.48)	8 (9.76)	1 (1.59)
Costs	5 (2.24)	3 (3.66)	2 (3.17)
No interest/resistance	3 (1.35)	5 (6.10)	
Lack of awareness/knowledge	9 (4.04)		
**No barriers**	36 (16.14)		18 (28.57)
**Non-codable**	29 (13.00)	10 (12.20)	7 (11.11)

See Supplementary Tables SII/SV/SVIII for the full list of the participants’ answers and the respective category.

**Table IV:** Reported desired support for implementing the guidelines.

	**ENDO** (*n* = 161), *n* (%)	**RPC** (*n* = 61), *n* (%)	**POI** (*n* = 46), *n* (%)
**Guidelines**			
Clearer and concise information	19 (11.80)	8 (13.11)	4 (8.70)
Better dissemination of the guidelines	7 (4.35)	2 (3.28)	2 (4.35)
Additional information	6 (3.73)	1 (1.64)	2 (4.35)
Patient version/leaflets/information	17 (10.56)	5 (8.20)	6 (13.04)
App/other digital formats	7 (4.35)	1 (1.64)	3 (6.52)
Translation/language	8 (4.97)	10 (16.39)	5 (10.87)
More graphic/pictorial information	5 (3.11)		
More supporting evidence	5 (3.11)		
Q&A by expert(s)	2 (1.24)		
Recommendations based on expert opinion			1 (2.17)
Updating			2 (4.35)
**Clinic**			
Endorsement of guidelines by local authority	1 (0.62)	3 (4.92)	
Funding	4 (2.48)		1 (2.17)
Equipment	2 (1.24)		
Management and staff receptivity	3 (1.86)		
Protocol implementation	2 (1.24)		
Specialized staff		1 (1.64)	
Multidisciplinary/team work		1 (1.64)	
**Staff**			
Time	3 (1.86)	3 (4.92)	1 (2.17)
Education/courses	15 (9.32)	6 (9.84)	
Motivational support	1 (0.62)	2 (3.28)	
**Patient**			
Education/awareness	4 (2.48)	2 (3.28)	1 (2.17)
Attitude/cooperation		1 (1.64)	
**Support not needed**	19 (11.80)	3 (4.92)	8 (17.39)
**Non-Codable**	31 (19.25)	12 (19.67)	10 (21.74)

See Supplementary Tables SIII/SVI/SIX for the full list of the participants’ answers and the respective category.

**Figure 3:**
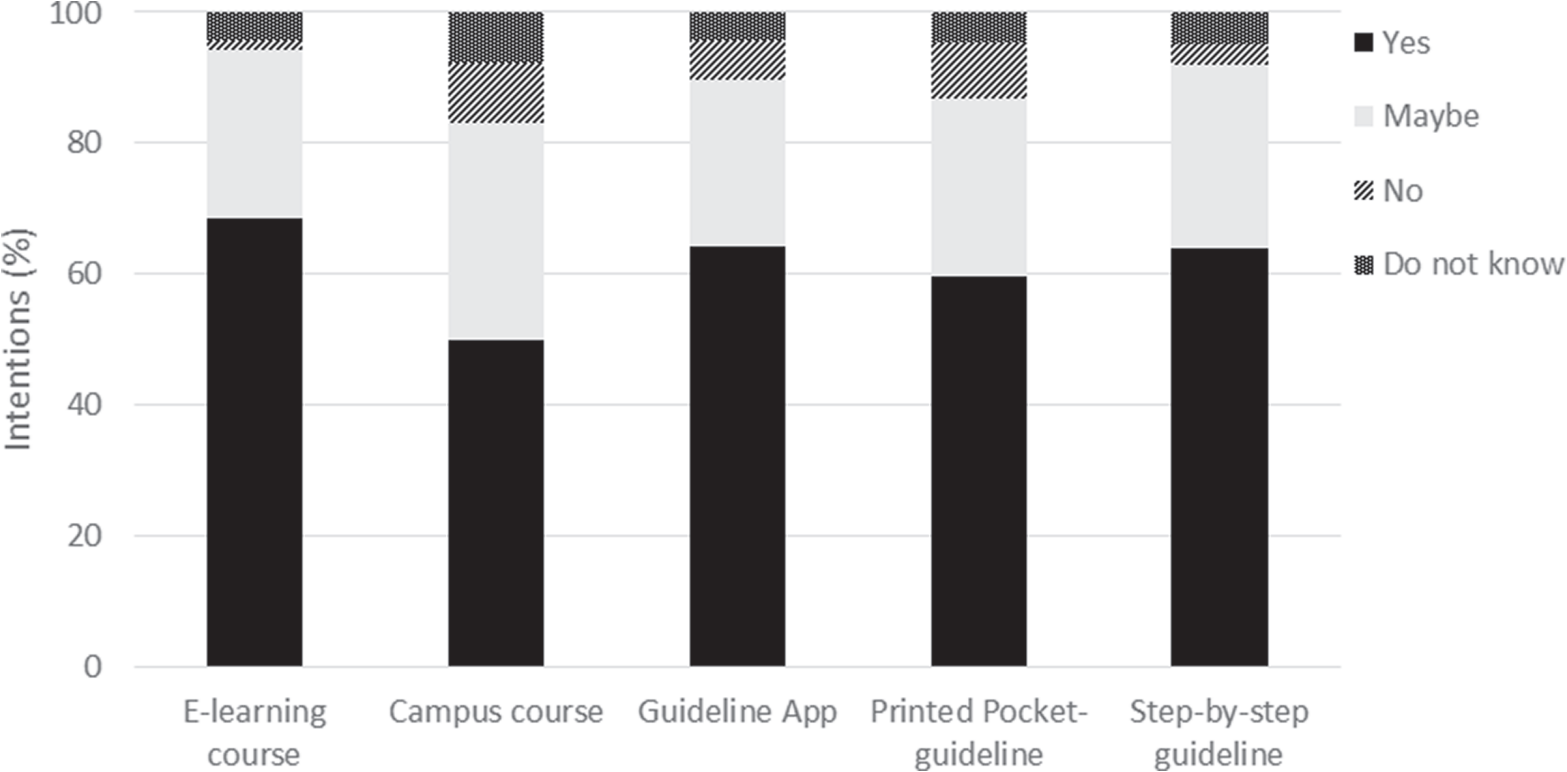
**Participants intentions to use different types of implementation support provided by ESHRE**.

Logistic regressions investigating the factors associated with knowing the ENDO guideline (χ^2^ = 11.777, *df* = 5, *P* = 0.038), downloading it (χ^2^ = 10.594, *df* = 5, *P* = 0.060), perceiving patient benefit (χ^2^ = 11.139, *df* = 5, *P* = 0.050) and referring them to the guideline (χ^2^ = 7.706, *df* = 5, *P* = 0.173) did not indicate any statistically significant association. The logistic regression showed a significant association with using the ENDO guideline in routine practice (χ^2^ = 86.718, *df* = 5, *P* < 0.001). Clinicians (OR = 14.69, 95% CI [7.22–29.91], *P* < 0.001) and those attributing a higher quality rating to the ENDO guideline (OR = 1.72, 95% CI [1.12–2.60], *P* = 0.011) were more likely to use it in their routine practice. The logistic regression showed a significant association with having made changes in their practice (χ^2^ = 15.315, *df* = 5, *P* = 0.009). European participants were less likely than others to have made changes in their practice (OR = 0.35, 95% CI [0.16–0.79], *P* = 0.011).

Logistic regressions investigating the factors associated with knowing the RPC guideline (χ^2^ = 9.375, *df* = 5, *P* = 0.095), downloading it (χ^2^ = 6.320, *df* = 5, *P* = 0.276), having made changes in practice (χ^2^ = 10.298, *df* = 5, *P* = 0.067) and perceiving patient benefit (could not be computed as all respondents indicated that they perceived benefit) did not indicate any statistically significant association. The logistic regression showed a significant association with using the RPC guideline in routine practice (χ^2^ = 15.170, *df* = 5, *P* = 0.010). Clinicians (OR = 3.68, 95% CI [1.37–9.86], *P* = 0.010) and those attributing a higher quality rating to the RPC guideline (OR = 2.58, 95% CI [1.17–5.67], *P* = 0.018) were more likely to use it in their routine practice. The logistic regression showed a significant association with referring patients to the RPC guideline (χ^2^ = 11.719, *df* = 5, *P* = 0.039). Older participants were less likely to refer their patients to the RPC guideline (OR = 0.92, 95% CI [0.87–0.98], *P* = 0.007).

Logistic regressions investigating the factors associated with knowing the POI guideline (χ^2^ = 7.441, *df* = 5, *P* = 0.190), downloading it (χ^2^ = 8.954, *df* = 5, *P* = 0.111), having made changes in practice (χ^2^ = 5.120, *df* = 5, *P* = 0.401), perceiving patient benefit (could not be computed as all respondents indicated that they perceived benefit) and referring them to the guideline (χ^2^ = 9.300, *df* = 5, *p* = 0.098) did not indicate any statistically significant association. The logistic regression showed a significant association with using the POI guideline in routine practice (χ^2^ = 33.495, *df* = 5, *P* < 0.001). Clinicians were more likely to use it in their routine practice (OR = 11.57, 95% CI [3.90–34.32], *P* < 0.001).

Finally, logistic regressions investigating the factors associated with knowing the RPL guideline (χ^2^ = 3.090, *df* = 5, *P* = 0.686), downloading it (χ^2^ = 2.666, *df* = 5, *P* = 0.751) and intending to make changes in routine practice (χ^2^ = 8.159, *df* = 5, *P* = 0.148) did not indicate any statistically significant association.

### Implementability of the guidelines

Figure 2 presents the quality ratings of the guidelines for the GLIA dimensions and overall. The average perceived quality of the four guidelines was higher than four (good quality; range 4.11–4.26) and ratings for the different GLIA implementability dimensions of the three guidelines tended to be slightly lower or higher than 4 (range 3.54–4.14).

### Perceived changes in practice due to implementing the guidelines

Table II presents the perceived changes in practice due to implementing the guidelines reported by 97 participants (total of 351 statements coded, reported in Supplementary Tables SI [ENDO], SIV [RPC] and SVII [POI]). Perceived changes were mostly related to what was reported as an improvement in the provision of care, either better treatment (e.g. ENDO, ‘guiding decision in patient’s treatment selection’, ‘deciding the most appropriate progestational therapy’; ‘improved safety/effectiveness of treatment’; POI, ‘offering hormone therapy’, ‘medicine dosage’, ‘proper treatment’), better psychosocial support/counselling (e.g. ENDO, ‘better counselling of endometriosis patients with infertility issues’; RPC, ‘offer more psychological support to patients having fertility treatment’, ‘how to handle those who do not get positive results’; POI, ‘began counselling of relatives on possible implications for them’) or overall better patient-centred care (e.g. ENDO, ‘shorter time for decision-making’; RPC, ‘refer patients at risk of emotional problems to specialised psychosocial care’, ‘cater to their psychosocial needs’; POI, ‘follow up of women with POI’). For the ENDO guideline perceived changes also reflected better screening/evaluation/diagnosis (e.g. ‘transvaginal sonography in diagnosis of rectal endometriosis’, ‘more consistency among clinicians in grading’) and better decisions about surgical treatment (e.g. ‘avoiding unnecessary surgery’, ‘feeling more confident about my decision to or not to operate an endometrioma’, ‘it helped me to take a decision about to make a laparoscopic intervention’) and treatment of pain (e.g. ‘it improved the trust the patient has in our practice by improving pain management’, ‘pain management’). For the RPC and POI guidelines, reported changes also related to an increase in awareness and knowledge (e.g. RPC, ‘more aware of the effect of the disease on the couple’, ‘be more aware of the patients’ needs and preferences’, ‘it makes me conscious of the importance of the information’; POI, ‘I was looking for literature and textbook for POI but this guideline is faster and easier to reach from just ESHRE website so time’, ‘well defined POI’). Finally, for the POI guideline, perceived changes also concerned screening/evaluation/diagnosis (e.g. ‘right prescriptions to find out a potential cause’, ‘clear and faster diagnosis’, ‘ruling out all possible causes of POI’).

### Perceived barriers to implementation of guidelines

Table III presents the perceived barriers to implementation of the guidelines reported by 94 participants (total of 368 statements coded, reported in Supplementary Tables SII [ENDO], SV [RPC] and SVIII [POI]). The most prevalent barriers common to the three guidelines were related to the guidelines themselves, more specifically, the lack of translation to languages beyond English (e.g. ENDO, ‘if it is only in English this would be a problem for some patients’; RPC, ‘only a small amount of my clients are fluent in English enough to appreciate the guidelines’; POI, ‘the language barrier is a bit difficult’), the perception that guidelines are too long and difficult to understand (ENDO, ‘to long to read and to apply in clinical practice’, ‘unclear recommendations’; RPC, ‘too large, needs short version of recommendation’, ‘not attractive to read: difficult and unclear layout at first sight’; POI, ‘very long’, ‘difficult to understand’) and lack supporting evidence (ENDO, ‘content validation’; RPC, ‘not enough evidence to make strong direct and practical recommendations to clinical staff’; POI, ‘limited evidence on topic’). Other common barriers were financial constrains (ENDO, ‘limited funding’; RPC, ‘cost effectiveness’; POI, ‘costs’) and lack of staff expertise (e.g. ENDO, ‘sometimes I feel myself in lack of clinical expertise which’ may be my limitation’; RPC, ‘experience’; POI, ‘lack of knowledge/expertise’) and time (e.g. ENDO, ‘it is more or less applicable in our clinic but lacking enough time to come back to the guidelines’; RPC, ‘no real time to do the promotion more than what we did’, POI, ‘time’). Patients’ cultural habits and norms, together with a lack of interest and resistance, were relevant barriers for the implementation of the RPC guideline (e.g. ‘find it difficult to co relate due to ethical differences’, ‘against local habits’, ‘patients are often refractory to psychological support’, ‘not cooperative’) and, to a lesser extent, the ENDO guideline (e.g. ‘incorrigible habits, sociocultural restraints’, ‘sometimes patients do not accept no surgery’). It should be noted that some participants reported not experiencing any barriers to the implementation of the ENDO and POI guidelines, but not the RPC.

### Perceived beneficial support for implementation of guidelines

Table IV presents the perceived beneficial support to implement the guidelines reported by 74 participants (total of 268 statements coded, reported in Supplementary Tables SIII [ENDO], SVI [RPC] and SIX [POI]). The most cited support was related to the guidelines themselves. Participants wanted clearer and more concise information on the guidelines (e.g. ENDO, ‘more clear overview, different layout that makes it more attractive and less confusing’, ‘short version’; RPC, ‘short crisp recommendation for quick reference’, ‘more concise written summary’; POI, ‘small flowchart to laminate in clinic’, ‘a quick summary’), better dissemination via multiple media (e.g. ENDO, ‘spread of guidelines to all health providers’, ‘summarized guidelines sent per email annually to all interested as a reminder’; RPC, ‘social media, Facebook, emails; POI, ‘guidelines must be spread through social media’) and translation to other languages (e.g. ENDO, ‘having the guidelines in different languages’; RPC, ‘multiple language translation with content validation’; POI, ‘a translated version’), as well as more patient-orientated materials (e.g. ENDO, ‘interactive online shared decision-making tool’, ‘simplified educational materials’; RPC, ‘leaflets for patients’, ‘an illustrated guide for patient’; POI, ‘podcast or small video guidance for patients’, ‘app’). Participants also reported wanting more education and courses on ENDO (e.g. to have a chance to attend ESHRE campuses’, ‘advocacy through seminar or workshop’) and RPC guidelines (e.g. ‘group teaching of all staff’, ‘e-learning course’). Figure 3 shows that most participants (60% or over) would use e-learning courses, apps, printed pocket guidelines and a step-by-step guide for implementation, if provided by ESHRE.

## Discussion

Results from this survey-based study show that ESHRE’s evidence-based guidelines are known and are being used worldwide. Guideline implementation is perceived to result in the improvement of treatment, better screening/evaluation/diagnosis and better psychosocial and patient-centred care (among other positive changes), leading to perceived benefits for patients. However, on average only 30% of those who know of the guidelines report implementing changes in their routine practice, and <20% think that patients benefit from it. Although the guidelines are perceived as highly implementable, the lack of translation, their non-friendly format (too long and complicated) and lack of supporting evidence are reported barriers to their implementation. Consistently, ESHRE members show willingness to use support for implementation, namely by doing online courses and using translated and engaging materials that provide a step-by-step approach to implementation.

More than 700 ESHRE members from all over the world (85 countries, 6 continents) accessed the online survey. The results indicate that the majority of ESHRE members know about and access the guidelines. Indeed, >80% of the respondents know the ENDO guideline, the first ESHRE guideline published in 2013, and almost 60% know the RPC, POI and RPL guidelines, the former two published in 2015 and the latter in 2018. Of those members who know the guidelines, around half downloaded at least one version of it (guideline document, *Human Reproduction* publication, pocket guideline, etc.). Results also indicate that the guidelines are being used equally inside and outside Europe, suggesting they have a wide geographical reach.

Overall, ESHRE members consider that the guidelines are of very high quality and fairly implementable; in other words, they perceive that it is possible to implement the guidelines at their clinics. Those who stated they used a guideline, report a wide array of perceived positive changes, mainly associated with the provision of better treatment, screening/evaluation/diagnosis and psychosocial and patient-centred care. The changes reported are expected and specific considering the recommendations of each guideline. For instance, participants considered that the ENDO guideline helped them to make better decisions about medical and surgical treatment of pain, that the RPC guideline increased awareness about and led to the provision of better psychosocial care, as well as more patient-centred care, and that the POI guideline facilitated the identification and treatment of patients with POI. Finally, on average, every three out of four people who made changes perceived that their patients benefited from it (ENDO 62%, RPC 80%, POI 75%). Overall these results suggest that, when used, the ESHRE guidelines have the potential to guide effective change at clinics.

Despite the potential to improve the quality of health care delivery, the current reality is that on average only one-fourth of those who are aware of the guidelines go on to make changes in their routine practice, and less than one-fifth perceive that their patients benefit from the guidelines. One possible explanation for these low implementation numbers may be that the guidelines are out of the professional remit of many participants. Indeed, the RPC guideline is the only one that targets all fertility staff members regardless of their professional background, while the other three guidelines are mainly written for clinicians. The results from the logistic regressions support this idea by showing that clinicians are more likely than other fertility staff members to use the guidelines in their daily practice. Supplementary Table SX reports the data on dissemination, implementation and impact for clinicians only. Percentages of use in daily practice are ~30% higher for ENDO, 16% for RPC and 34% for POI, showing that clinicians are indeed more likely to use the guidelines. Another possible reason for the reported low implementation rates is that respondents might already be following the guidelines and therefore did not need to make any additional changes. We explored this possibility by analysing data from an open-ended question (data not shown) where participants were given the opportunity to justify the answer given to the question if they or the clinic had made changes in their routine practice. These data showed that on average 1–3 out of each 10 participants were already following the guidelines (ENDO, 28.0%; RPC, 19.0%; POI, 19.2%; RPL, 11.4%). Overall these data show that one should not expect 100% implementation rates because not all respondents are in a position where they should be applying the guidelines and some are already applying them. If we look at implementation considering the groups targeted by each guideline (physicians for ENDO, POI and RPL; and all fertility staff for RPC) then rates of use in daily practice vary between 29.5% (RPC) and 72.1% (ENDO) and rates of participants who made changes vary between 18.9% (RPC) and 55.4% (ENDO), providing a more optimistic view of implementation for all guidelines but the RPC.

Nevertheless, the data also indicate that the guidelines are not being implemented because of perceived barriers. First, results from logistic regressions suggest that perceived lack of quality of the guidelines is a barrier to their use in routine practice. Second, results from qualitative analysis show that participants identified other barriers that were common to the three guidelines investigated: the lack of translated versions (beyond English) and the lack of brief, friendly and engaging formats were reported most frequently. The long and difficult format has been reported frequently in other domains (Cabana *et al*., 1999; Cochrane *et al*., 2007; Haagen *et al*., 2005). This is understandable considering that fertility staff feel highly work- and time-pressured (Boivin *et al*., 2017) that some members of staff (e.g. clinicians) need to familiarize themselves with all the guidelines and that many have limited English proficiency. Contrary to what was expected and has been previously described (Cabana *et al*., 1999; Cochrane *et al*., 2007; Haagen *et al*., 2005), local barriers (i.e. clinic, staff and patient related) seemed to be less relevant to implementation. The most significant ones were lack of financial resources (clinics and patients), time and knowledge/expertise. In the specific case of the RPC guideline, cultural and normative factors also seemed to be relevant, which may reflect the prevalence of a more medically orientated approach to fertility treatment, as opposed to a more holistic patient-centred one, whereby patient well-being and quality of life is highly valued by staff and patients alike as an important treatment quality indicator (Picker Institute, 2012, World Health Organization, 2007). It may be that respondents focused more on barriers that they thought ESHRE could tackle, that is, those directly related with the guidelines. It may also be that the lower reporting of local barriers is associated with the smaller percentage of people that are actually using the guidelines and therefore come across these barriers.

Overall these results suggest that ESHRE’s current policy of investing in the guidelines’ methodological quality and dissemination is important, but insufficient to ensure their full implementation at clinics. On the bright side, results suggest that it is feasible for ESHRE to address some of the barriers to implementation, as most reported barriers were related to the guidelines rather than the clinic, staff or patient. Results on requested support for implementation further reinforce this idea, as these focus on accessing brief and engaging materials (for staff and patients), translated versions of the materials and (online) training courses that provide clear instructions for implementation. To a certain extent, ESHRE is already addressing these requests (e.g. presentations on the guidelines at campus courses, pocket versions of some documents), but the emphasis has been on communicating *what* is recommended for staff to do. In the future ESHRE should put the emphasis on *how* to do it at clinics and should try to ensure a positive balance between learning time required and implementation skills acquired for all guidelines-related educational activities.

ESHRE has invested heavily in developing evidence-based guidelines to improve health care delivery across Europe. It is timely and necessary to adopt the same evidence-based approach to understand how well the guidelines are being disseminated and implemented as well as their impact on patients. The current online survey allowed us to develop a comprehensive picture of how the ESHRE community views and uses the guidelines, with detailed information on which changes were perceived to have been implemented plus any barriers and desired support for implementation. However, some limitations need to be acknowledged. The survey was limited to ESHRE members and therefore not representative of the European population of fertility health care professionals, which may be less sensitive to the benefits of evidence-based practice, less up-to-date on recent developments in the field and have lower English proficiency than respondents in this survey. The survey was very long and required participants to provide the same information for each of the four guidelines in a consecutive way (ENDO, RPC, POI, RPL). This resulted in an increasing number of missing data as we progressed through the guidelines answers. It is unclear if missing data result from response fatigue or lack of interest in the guidelines. Finally, the survey assessed participants’ perceptions and not their actual practice at their clinics. The implication of these limitations is that the results reported may be too optimistic and may only capture the views of those who actively engaged in trying to implement the guidelines.

In sum, implementation tools for guidelines are important, although there is very little evidence for their efficacy (Flodgren *et al*., 2016). The survey’s results provide some clear input on the preferences of users regarding implementation tools for the ESHRE guideline and suggest that ESHRE should, first, consider investing in the translation of the guidelines and all associated materials into different languages. Second, ESHRE should continue to develop new engaging ways of disseminating the guidelines to different stakeholders (staff and patients). Using different media formats seems to be valued by participants but it poses additional challenges in terms of costs in production and translation. Finally, ESHRE should consider investing in online education and training focused on a ‘hands-on’ step-by-step approach to implementation. The combination of these three strategies should constitute an important step forward in addressing barriers to implementation. The barriers and support requests were common to the three guidelines; therefore support activities that proved to be efficient with one guideline could be easily transferred to the remaining guidelines. Overall, these efforts should bring ESHRE closer to improving the quality of fertility health care across Europe.

## Authors’ roles

S.G. and N.V. designed the study and online survey. All authors were involved in data analysis and writing and final approval of the manuscript.

## Funding

No funding was received for this study.

## Conflict of interest

S.G. and N.V. are involved in the development and dissemination of the guidelines. M.S.L. has no conflicts of interest.

## Supplementary Material

Supplementary_Tables_final_hoz011
