# Supplementary material for: Dissemination, implementation and impact of the ESHRE evidence-based guidelines
Source: Hum Reprod Open. 2019 Jun 6;2019(3):hoz011. doi: 10.1093/hropen/hoz011 (PMC6561327; doi:10.1093/hropen/hoz011)
Supplement: Supplementary_Tables_final_hoz011 [file supplementary_tables_final_hoz011.docx]

| **Supplementary Table SI**  Changes in practice due to applying the Endometriosis guideline reported by participants. | |
| --- | --- |
| **Participants’ answers** | **Category** |
| Teaching of postgraduate trainees | **Increased awareness/knowledge** |
| Discussed in journal club |  |
| Awareness of female healthcare |  |
| Awareness |  |
| Support my clinical thinking |  |
| Helped clinic staff to understand endometriosis better. |  |
| Diagnosis of ovarian endometrioma in premenopausal women on the following ultrasound characteristics: ground glass echogenicity and one to four compartments and no papillary structures with detectable blood flow | **Better screening/evaluation/diagnosis** |
| Transvaginal sonography in diagnosis of rectal endometriosis |  |
| Diagnosis |  |
| Diagnosis |  |
| Meticulous history |  |
| Offer diagnostic laparoscopy in unexplained infertility to look for minimal to mild endometriosis which can be ablated |  |
| Diagnosis |  |
| Diagnostics |  |
| Screening and investigations |  |
| Decision in the diagnosis and |  |
| Diagnostic evaluation |  |
| Proper classification |  |
| Biomarkers in endometrial tissue, menstrual or uterine fluids to diagnose endometriosis are not recommended |  |
| Ultrasound in women with ruling out rectal endometriosis |  |
| Proper investigations |  |
| Measurement of AMH and AFC before and after chirurgical treatment |  |
| 3D ultrasound |  |
| More consistency among clinicians in grading |  |
| Proper investigations |  |
| Immunological biomarkers, including CA-125, in plasma, urine or serum to diagnose endometriosis are not recommended |  |
| Expedite referral for first consultation In cases of endometriosis |  |
| End CYSTS | **Better general treatment** |
| Expediting patients for IVF |  |
| Proper treatment strategies |  |
| Endometriomas management |  |
| Higher threshold for performing a laparoscopy before IVF |  |
| I prefer ivf for recurrent endometriosis |  |
| Management of endometriosis in relationship with fertility |  |
| Tailor preparation for IVF |  |
| Don't use coc pt endometriosis if was to pregnant |  |
| I know exactly wat is more important for patient. Surgery or conservative treatment |  |
| Diminish laparoscopies before IVF |  |
| Ivf and endometriosis |  |
| Freeze all if it's chocolate cells were seen |  |
| Time saving |  |
| Increased effectivenes of treatment |  |
| Guiding decision in patient's treatment selection |  |
| IVF Treatment |  |
| Addressing DIE more effectively |  |
| IVF vs Videolaparoscopy choise |  |
| Time to ART |  |
| Time to intervention |  |
| Endometrioma management |  |
| Management of infertility |  |
| Management of infertility |  |
| Endometrioma management |  |
| Use gnrh analog for pretreatment ivy , not to only decreased the cyst |  |
| Recommend operation prior to IVF |  |
| Intrauterine insemination in women with endometriosis |  |
| Which best therapy |  |
| IVF with present ovarian endometrioma |  |
| Indication of IVF treatments |  |
| Try iui after 3-6 mo don't success |  |
| Deciding infertility approaches |  |
| Expedite Ivf treatment when waiting times long |  |
| How long to wait for natural conception after operation |  |
| Previous treatment before IVF |  |
| More evidence based decisions |  |
| Reduced endometrioma size |  |
| Endometriotic cyst aspiration prior to IVF in those with recurrent cyst after cystectomy |  |
| Management during ivf |  |
| Medical therapy and infertility |  |
| Improved safety of treatment |  |
| Therapy time |  |
| Treatment |  |
| Do or not surgery prior to IVF |  |
| Using special media to improve the quality like calcium |  |
| Priority for the treatment |  |
| Planning treatment |  |
| Deciding the most appropriate progestational therapy |  |
| Antibiotic prophylaxis for oocyte pick up |  |
| Reason for IVF |  |
| Assisted reproductive technology |  |
| Sugesting management |  |
| Düring of treatment |  |
| Could choose the right treatment |  |
| Surgery prior ART with endometriomas |  |
| Fertility treatment |  |
| More ivf without previous surgical treatment |  |
| Planning IVF treatment with endometriomas |  |
| Don't waste time before fertility treatment |  |
| For controlled ov stimulation and iui vs Oi |  |
| Right treatment modes |  |
| IVF is the best option for women with endometriosis |  |
| Long agonist protocol for IVF patients |  |
| Improved safety of treatment |  |
| Therapy time |  |
| Treatment |  |
| Protocols used for ivf treatment |  |
| Decreased time |  |
| Treatment with endometriomas |  |
| Duration of the treatment |  |
| Gnrh agonist applications | **Better medical treatment of pain** |
| said / coc to treat endometriosis w/ pain |  |
| Treatment of pain in extragenital endometriosis |  |
| Reduced pelvic pain |  |
| When to give medical management in endometriosis |  |
| Management of pain |  |
| Reason for hormonal therapy |  |
| Hormonal therapies |  |
| Hormonal therapy |  |
| When to do pharmaceutical treatment |  |
| Medical treatment |  |
| Medical Treatment |  |
| More medical treatment |  |
| Hirmolan therapy |  |
| It improved the trust the patient has in our practice by improving pain management in our patients |  |
| Pain |  |
| Pain management |  |
| Pain management |  |
| Adjuvant therapies |  |
| Special medication also for the patient |  |
| Avoiding unnecessary surgery | **Better surgical treatment** |
| Not offering GnRH pre-op or post-op |  |
| Clarity on surgical management of endometriosis |  |
| Planning surgeries |  |
| Only surgery in highly specialised centers |  |
| Reason for operation |  |
| Surgery |  |
| Surgery |  |
| Relaparoscopy |  |
| It helped me to take a decision about to make a laparoscopic intervention |  |
| Surgical indications |  |
| When to do operation |  |
| Not operating endometriomas |  |
| Deciding of which patient to operate |  |
| Reduction in referral for surgery |  |
| Don’t do cystectomy for cases of choclate cyst less than 3 cm |  |
| No surgery for endometriomas less than 3 cms |  |
| When to operate endometriosis |  |
| Less surgical treatment |  |
| Reducing surgical intervention |  |
| On when to operate |  |
| Decision for repeat surgery |  |
| Less operating |  |
| Avoid the chirurgical treatment in patients with low ovarian reserve |  |
| Postpone the laparoscopy in small chists |  |
| Think a lot more about surgery decision |  |
| Fewer unnecessary surgical interventions |  |
| Surgery |  |
| Less indications for laparo |  |
| Not to operate for pain which is related with endometriosis |  |
| Excision rather than drainage of endometrioma |  |
| Only remove endometriomal >3cm |  |
| No surgery on endometriomas if the patient has none or mild symptoms |  |
| Unilateral cyst if patient is asymptomatic is left alone |  |
| Size of endometrioma |  |
| Ovarian reserve test for patient. Before the ERA of AMH it was not taken into consideration and all endometriomas were resected |  |
| Cystectomy better than ablation |  |
| I can use 2 or 3 stap treatment for endometrioma |  |
| Don't perform cystectomies unless necessary |  |
| Decision ao surgery for endometrioma size |  |
| Feeling more confident about my decision to or not to operate an endometrioma |  |
| Avoiding complication of surgery |  |
| Surgery approach |  |
| Surgical management |  |
| Laparoscopy with ablation or resection the best treatment for endometriosis gr 1-2 |  |
| Ovarian reserve after operation |  |
| Electrosurgery selection |  |
| Surgical treatment |  |
| Fulgration of minimal to mild endometriosis improve outcome |  |
| How to treat cysts of endometriosis |  |
| Correct surgical technique |  |
| No GnRH before laparoscopy |  |
| Surgery for patient with endometriosis and infertility |  |
| It enabled us to know when to refer patients with moderate to severe endometriosis for patients who need surgery to enable then attain quality care from a specialist |  |
| Psycological support | **Better psychosocial support/counselling** |
| Evidence based counselling |  |
| Better quality of patient counseling |  |
| Patient counselling |  |
| Adjunct in counselling patients of their treatment options |  |
| Better counselling of endometriosis patients with infertility issues |  |
| Shorter time for decision making | **Better patient-centred care** |
| Clinical management |  |
| clinical management |  |
| Management of women with endometrioma |  |
| Unified approach to patient with endometriosis |  |
| Decision-making |  |
| Affected on the quality occurred |  |
| Management |  |
| Better clinical results |  |
| Non pharmacolgical management |  |
| Follow up of patients with endometriosis |  |
| I can apply patient to save their fertility potential |  |
| Providing useful information | **Better education/information provision** |
| More information available to patient |  |
| More information about endometrioses |  |
| It improved our knowledge on the practice to give better quality care to our patients and enable them make informed choice |  |
| Now I am more structured in answering and clarifying patients' questions about endometriosis |  |
| Better quality of patient education |  |
| Could explain well to the patient |  |
| More indications for lifestyle change |  |
| More confidence to explain the clinical approach |  |
| Explaining infertility patients that surgical and not medical treatment is the recommendation |  |
| Guidelines on lifestyle change and behavior |  |
| Postoperatively preventive aspects for preventing recurrence are explained to patient |  |
| I explain my patients about eshre proposals on endometriosis |  |
| Educating spouses and in laws your lady needs her healthcare as a right |  |
| Reliable information for patient |  |
| More information towards patients |  |
| Source of solid evidence for patient information |  |
| Educate |  |
| More indications for lifestyle change |  |
| Advocacy about endometriosis | **Endometriosis advocacy** |
| Advocacy |  |
| Provided a basis for audit | **Clinical auditing** |
| Decrease cost for treatment | **Reduced costs** |
| Cost reduction |  |
| Infertility patients | **Non codable** |
| Systematic |  |
| Step by step considering in a methodology |  |
| Updated information |  |
| First line therapy |  |
| We approach patient change |  |
| Incidental endometriosis |  |
| Evidence based studies |  |
| Broad panel ofxperts |  |
| Selection |  |
| Pregnancy delay |  |
| Easy work |  |
| Decreased recurrence |  |
| Beneficial |  |
| It was Evidence based, so can be applied |  |
| Selection |  |
| Take home baby |  |
| Patient response |  |
| Trust |  |

**Note:** The entries in all supplementary tables (SI-SX) are exactly how participants wrote them i.e.these are the raw data and have not been changed.

| **Supplementary Table SII**  Barriers to implement the Endometriosis guideline reported by participants. | |
| --- | --- |
| **Participants’ answers** | **Category** |
| **Guidelines** |  |
| Not enough understandable | **Unclear/difficult to understand** |
| Unclear recommendations in some cases |  |
| Unclear |  |
| Examples are unclear |  |
| Scattered evidence |  |
| Eхamples are unclear recommendations |  |
| Unclear layout, kind of confusing impression about layout |  |
| Too large | **Too long** |
| Large aspects |  |
| Large, too many sessions |  |
| Lengthy |  |
| To large |  |
| Guideline is too large |  |
| Guideline is a bit large and hard to read |  |
| Too large |  |
| It is very big |  |
| They are a little extensive |  |
| Guideline is too large |  |
| Maybe too large |  |
| Is to large |  |
| Too large |  |
| To long to read and to apply in clinical practice |  |
| Very large |  |
| Guideline is large |  |
| Guideline is very large |  |
| Limited evidence | **Lack of evidence** |
| Limited evidence on the topic |  |
| Limited evidence (Clinicians may consider both ablation and excision of peritoneal endometriosis to reduce endometriosis-associated pain) |  |
| Too many inconclusive situations |  |
| Some have lack of grade 1 evidence |  |
| Limited evidence - based on primitive models |  |
| Limited evidence |  |
| Limited evidence in some cases, eg endometriosis and IVF |  |
| Content validation |  |
| Limited evidence |  |
| Language | **Language/translation** |
| Need language translation |  |
| If it is only in English this would be a problem for some patients - moved from patient |  |
| No Dutch version - moved from patient |  |
| Language - moved from patient |  |
| They speak German, few speak Englisch - moved from patient |  |
| Lack of patient knowledge due to language barrier (not all patients can understand English) - moved from patient |  |
| Need to translate the content to local language - moved from patient |  |
| Wrong interpretations and misunderstandings, language - moved from patient |  |
| Language - moved from patient |  |
| Inadequate | **Topic not relevant/inappropriate/not priority** |
| Many other societies prefer nice or sign guidelines so not recommend - moved from clinic |  |
| No new information as we already used - moved from clinic |  |
| We have our local patient resources on endometriosis |  |
| NICE guideline exists |  |
| Must more specific | **Lack of specific information** |
| Treatment for moderate endometriosis for fertility and pain |  |
| Diverse guidance, often no specific direction |  |
| Some points remains controversial, otherwiese the guidelines are really good |  |
| Unclear recommendations on difficult clinical situations connected with ovarian endometriomas |  |
| Challenging to tailor guideline recommendations to individual patients - moved from staff |  |
| Difficult to understand - moved from patient | **Lack of patient friendly version/materials** |
| Easy information - moved from patient |  |
| Difficult to understand - moved from patient |  |
| Not easy to understand - moved from patient |  |
| Patient frindly leaflets missing - moved from patient |  |
| Guidelines can be disseminated through social media like Facebook. | **No dissemination/awareness** |
| Lack of power | **Lack of power** |
| Lack of power |  |
| Lack of power |  |
| Lack of power |  |
| Lack of power |  |
| **Clinical setting & system** |  |
| Costs | **Costs/financial constrains** |
| Costs - moved from GL |  |
| Cost |  |
| Costs |  |
| Cost |  |
| Costs |  |
| Costs associated for diagnosis of deep endometriosis |  |
| Cost (use of a levonorgestrel-releasing intrauterine system (LNG-IUS) |  |
| Limited funding |  |
| Costs |  |
| Costs |  |
| Cost |  |
| Costs of course is a problem especially laser surgery |  |
| MRI is a costly investigation; Doing laparoscopy in a country that is funded only by the patient will be difficult |  |
| Expensive |  |
| Costs |  |
| Waiting list to surgery | **Lack of infrastructure/equipment** |
| Lack of facilities |  |
| Equipment |  |
| Different clinical resources e.g equipments |  |
| Need for 3D scan/MRI |  |
| Need more specific equipment |  |
| Need for specific equipment |  |
| Need more equipment and new equipment |  |
| Need for specific equipment |  |
| Need to be connected | **Lack of trained/specialised staff** |
| We are only offering fertility treatment, not treatment of endometriosis |  |
| Need for well-educated staff |  |
| Availability of healthcare provider - moved from staff |  |
| Routine practice, director's reluctancy | **Culture/norms** |
| Clinics are big organisms and it is difficult to settle on the same thing |  |
| Different setting with our local conditions - moved from Guidelines |  |
| **Staff** |  |
| Lack of expertise | **Lack of knowledge/expertise** |
| Lack of knowledge |  |
| Lack of expertise |  |
| Expertise |  |
| Lack of knowledge |  |
| Lack of knowledge (presacral neurectomy (PSN) is effective as an additional procedure to conservative surgery to reduce endometriosis-associated midline pain, but it requires a high degree of skill and is a potentially hazardous procedure |  |
| Lack of expertise, need to improve awareness of guidelines |  |
| Lack of knowledge |  |
| Personal skills and lack expertise and standardise the treatments for all clinicians |  |
| Surgical expertise/ lack of consensus |  |
| I did not know there was a patient version. |  |
| Lack of expertise |  |
| Sometimes I feel myself in lack of clinical expertise which may be my limitation |  |
| Need more personal and more knowledge |  |
| Ability to explain the subject for patients |  |
| See above (Need to be read, understood and discussed by all staff involved) |  |
| Need to be read, understood and discussed by all staff involve - moved from GL |  |
| See above (Need to be read, understood and discussed by all staff involved) - moved from clinic |  |
| Wide spread knowledge and uniform practice is lacking |  |
| Time | **Lack of time** |
| Time to get the investigation reports |  |
| Time |  |
| No barrier, just time |  |
| Limited time for implementing new things |  |
| Limited time for implementing new things - moved from clinic |  |
| It is more or less applicable in our clinic but lacking enough time to come back to the guidelines - moved from clinic |  |
| Time constraints - moved from patient |  |
| Lack of interest | **Lack of interest/motivation** |
| Irrelevant |  |
| Persistence od a old habit | **Culture/norms** |
| Tradition |  |
| Decades of personal experience |  |
| **Patient** |  |
| Ready to wait and take painkillers | **Culture/norms** |
| Incorrigible habits .socioculture restraints. |  |
| Unable to give history shy or overprotected by in laws. |  |
| Local habits and lack of trust |  |
| Local habits |  |
| Patients are non compliant to oral contraceptives(daily pill) |  |
| Against local habits |  |
| fear of the new guidelines |  |
| Patients do not believe in science and recommendations |  |
| Local habit |  |
| Patient reject because the cost to expensive | **Costs** |
| Cost of procuring the medicines from overseas |  |
| Patients have problems with the costs in our country |  |
| Cost limitations |  |
| In Mexico insurance does not cover endometriosis besides |  |
| Patients sometimes search for different kind of clinicians and if they did not get information from these guidelines patines are hard to convince them | **No interest/resistance** |
| Sometimes patients do not accept no surgey |  |
| Most of the patients come with an idea in their minds generally from trash resources from internet so it is difficult to persuade |  |
| Lack of education | **Lack of awareness/knowledge** |
| Comprehension |  |
| Awareness with regard to the disease |  |
| Lack of knowledge |  |
| Explaining about the need for laparoscopy becomes difficult in unexplained infertility |  |
| Low education level, |  |
| Lack of knowledge of medical insight to grasp healthcare information |  |
| Patients often feel overwhelmed by the amount of information they are given and expected to absorb |  |
| lack of education |  |
| **No barriers** |  |
| None | **No barriers** |
| No |  |
| I really think the guideline is very useful |  |
| NA |  |
| Without barriers. |  |
| No |  |
| N/A |  |
| None |  |
| None |  |
| I really think the guideline is very useful |  |
| NA |  |
| Nothing specific |  |
| Same (without barriers) |  |
| No barrier |  |
| None |  |
| No |  |
| N/A |  |
| None |  |
| None |  |
| I really think the guideline is very useful |  |
| NA |  |
| None |  |
| Nothing specific |  |
| No barrier |  |
| No |  |
| N/A |  |
| None |  |
| None |  |
| No |  |
| I really think the guideline is very useful |  |
| NA |  |
| Nothing specific |  |
| None |  |
| I do not know |  |
| N/A |  |
| Empowered patients make informed decisions,at my clinic ART is mostly private and many patients will also pay privately for appropriate care not provided by the NHS, the guideline gives fabulous advice in an easy to understand way - it's super! |  |
| **Non codable** |  |
| Laparoscopy ablation for gr 1-2 endometriosis | **Non codable** |
| Endometriosis |  |
| Guideline on hormonal treatment |  |
| See answer at 8 (Occasionally, as most endometriosis patients are seen by a colleageau who specialises in endometriosis) |  |
| Patients are eager for improvements |  |
| Nil |  |
| Guideline itself is easy to comprehend |  |
| Gyanecology |  |
| Available for laparoscopy |  |
| The Comforters place specialist medical clinic |  |
| Only at tertiary care |  |
| See answer at 8 (Occasionally, as most endometriosis patients are seen by a colleageau who specialises in endometriosis) |  |
| Very versatile preferences |  |
| Must adhere to guide as much as possible |  |
| Consultant |  |
| Plan laparoscopy |  |
| Availability of the brands of medicines used in medical dura |  |
| Dr gbenga/ Dr ladipo |  |
| Same |  |
| See answer at 8 (Occasionally, as most endometriosis patients are seen by a colleageau who specialises in endometriosis) |  |
| Implementation remaind difficult |  |
| Fertility patient |  |
| The therapy conditions is not totally new |  |
| Mrs Esi- Rowland |  |
| In infertile women with endometriosis, clinicians should not prescribe hormonal treatment for suppression of ovarian function to improve fertility |  |
| Varied demographics |  |
| Its not usefull for them |  |
| See answer 8 (Occasionally, as most endometriosis patients are seen by a colleageau who specialises in endometriosis) |  |
| I have no patients |  |

| **Supplementary Table SIII**  Support to implement the Endometriosis guideline reported by participants. | |
| --- | --- |
| Participants’ answers | Category |
| **Guidelines** |  |
| Quick references card | **Clearer and concise information** |
| Tables |  |
| Clear instructions on surgical treatment and use of ART |  |
| Table format of treatment and medication similar to ADA guideline on diabetes therapy progession |  |
| Summery |  |
| Shorter |  |
| Specific |  |
| More concrete and concise |  |
| Small flowchart to laminate in clinic |  |
| Short version |  |
| Shorter |  |
| More clear overview, different layout that makes it more attractive and less confusing |  |
| Summary of information |  |
| Quick references - moved from clinic |  |
| Shorter - moved from clinic |  |
| Quick references - moved from staff |  |
| Shorter - moved from staff |  |
| Shorter - moved from patient |  |
| Easy information - moved from patient |  |
| Summarized guidelines sent per email annually to all interested as a reminder (just one A$-Paper) | **Better dissemination of the guidelines** |
| Spread of guidelines to all health providers |  |
| Spread it through whole clinicist community |  |
| ESHRE should liase with Obstetrics and Gynecology society of India to make the clinicians aware of the guidelines - moved from staff |  |
| Get closer through social media (some graphics on Instagram, Facebook) - moved from patient |  |
| Guidelines must be accessible from much more internet sites - moved from patient |  |
| It is a tough issue, maybe more promotion in the local societies will work - moved from clinic |  |
| Recommendations on who they are directed towards | **Additional information** |
| More detail explanation |  |
| The detailed information |  |
| Very specific recommendations, i.e. clinical cut-off values and diagnostic criteria |  |
| More precise and to the point |  |
| Individualised treatment - moved from patient |  |
| Information sheets - moved from patient | **Patient version/leaflets/information** |
| Leaflet - moved from patient |  |
| App - moved from patient |  |
| Interactive online shared decision making tool e.g. OptionGrid - moved from patient |  |
| To be more leaflets about endometriosis and fertility potential - moved from patient |  |
| Having patient info sheet in - moved from patient |  |
| Leaflet - moved from patient |  |
| More patient friendly aaps - moved from patient |  |
| patient friendly reading material with case stories and connecting to an endometriosis support group - moved from patient |  |
| Easy information - moved from patient |  |
| A reliable and serious guide - moved from patient |  |
| Laymans terms - moved from patient |  |
| Simplified educational materials - moved from patient |  |
| Perhaps a simplified version for patients - moved from patient |  |
| Providing meaningful and interpretable date - moved from patient |  |
| Patient app - moved from patient |  |
| Good information provided to the patient - moved from patient |  |
| Maybe a app | **App/other digital formats** |
| Easily accessible applications |  |
| Digital support |  |
| Podcast or small video guidance for patients - moved from clinic |  |
| Digital - moved from clinic |  |
| App |  |
| App |  |
| Translation to local languages - moved from staff | **Translation/language** |
| Having the guidlines in different languages - moved from patient |  |
| Sutch versions - moved from patient |  |
| Moré langu - moved from patient |  |
| Multi-language translations for patient contents - moved from patient |  |
| Multi-language translations for patient contents - moved from patient |  |
| local language - moved from patient |  |
| Patient version on Russian language would be helpful - moved from patient |  |
| More pictorial to overcome language barrier | **More graphic/pictorial information** |
| More graphics - we can show them and better explain to the patients |  |
| Hanging posters or table flip cards. - moved from clinic |  |
| Flow diagrams |  |
| Flow chart - moved from clinic |  |
| Multi centric data | **More supporting evidence** |
| More studies would clarify some recommendations |  |
| More of level 1 evidence |  |
| I think more beneficial would be larged evidenced based Guidelines |  |
| The power of evidence - moved from staff |  |
| Expert panel group who will be answering queries | **Q&A by expert(s)** |
| Consultant - moved from staff |  |
| **Clinic** |  |
| Support from our local organisations and pharmaceutical companies - moved from patient | **Endorsement of guidelines by local authority** |
| Lesser costs of surgery | **Funding** |
| More government funding; institutional funding |  |
| More funding |  |
| Help with costs |  |
| More equipments | **Equipment** |
| Equipment for laparoscopic surgery |  |
| Interest for the clinic side | **Management & staff receptivity** |
| Management awareness |  |
| Clinical staff willing to read, understand and discuss the recommendations |  |
| Implementation of guidlines as local treatment protocol | **Protocol implementation** |
| Controlling the implementation of guidlines as local treatment protocol |  |
| **Staff** |  |
| Time | **Time** |
| Time - moved from GL |  |
| Time - moved from clinic |  |
| Training | **Education/courses** |
| More team working to know what the consultants are doing - moved form clinic |  |
| Do it several clinics together - moved from clinic |  |
| Trainings for stuff - moved from clinic |  |
| More training |  |
| Sharing expert clinical opinion |  |
| Personel experience is also very important bu guidelines can also guide some gray zones about experiences also |  |
| Willing to read, understand and discuss the recommendations |  |
| Debate with clinicians and other users (to compare observations and conclusions) |  |
| Create more awareness |  |
| Health education |  |
| Short courses or workshops to improve expertise |  |
| Advocacy through seminar or workshop |  |
| To have a chance to attand ESHRE campuses. |  |
| Encouraging annula national meeting in every country for endometriosis experts - moved from clinic |  |
| Interest | **Motivational support** |
| **Patient** |  |
| Encouraging patient education - moved from clinic | **Education/awareness** |
| Cooperation better if awareness |  |
| Inteligence to understand |  |
| More health education |  |
| **Support not needed** |  |
| None | **Support not needed** |
| N/A |  |
| The way it is! |  |
| None |  |
| None |  |
| N/A |  |
| No support is needed |  |
| The way it is! |  |
| None |  |
| None |  |
| None |  |
| N/A |  |
| No support is needed |  |
| The way it is! |  |
| None |  |
| None |  |
| N/A |  |
| The way it is! |  |
| I do not know |  |
| **Non codable** |  |
| More generalizability | **Non codable** |
| Clinical skills are for expertising the disease |  |
| See aswer at 8 (Occasionally, as most endometriosis patients are seen by a colleageau who specialises in endometriosis) |  |
| Guidelines are very helpful |  |
| Strip 80–90% of the cyst wall and perform a partial cystectomy, as described above, up to the ovarian hilum. Laser, plasma energy, or bipolar can then be applied to treat the remaining endometriotic tissue (10–20%). |  |
| The extensive review and evidence-based medicine |  |
| For discussion about endoemtrioma management |  |
| See aswer at 8 (Occasionally, as most endometriosis patients are seen by a colleageau who specialises in endometriosis) |  |
| The comforters place |  |
| Clinic evidence |  |
| Reproductive medicine |  |
| Yes |  |
| Help you make decision |  |
| We can download and print if Esther cannot send such materials |  |
| Irrelevant |  |
| See answer at 8 (Occasionally, as most endometriosis patients are seen by a colleageau who specialises in endometriosis) |  |
| Don't do to much for cystectomy |  |
| Dr gbenga |  |
| Personal especie for several clinics |  |
| As above |  |
| Personal |  |
| See answer 8 (Occasionally, as most endometriosis patients are seen by a colleageau who specialises in endometriosis) |  |
| Patient history about fertility and ovarian reserve for guiding |  |
| Mrs Esi-Rohwland |  |
| Time and patient |  |
| Awareness |  |
| I don’t have patients |  |
| Fertility patient |  |
| As above |  |
| Patient page is satisfactory |  |
| Resolve concerns |  |

| **Supplementary Table SIV**  Changes in practice due to applying the Routine Psychosocial Care guideline reported by participants. | |
| --- | --- |
| **Participants’ answers** | **Category** |
| Better knowledge of these problems | **Increased awareness/knowledge** |
| Realised (lack of) evidence for intervention |  |
| It makes me conscious of the importance of the information |  |
| Awareness/consciousness of the psychosocial wellbeing of patients |  |
| Be more aware of the patients' needs and preferences |  |
| Realised lack of tools for couples after treatment |  |
| Being able to anticipate more on that aspect of patient |  |
| More aware on the effect of the disease on the couple |  |
| Helped me better understand psychological aspects of patients with infertility |  |
| Counseling is essential |  |
| Realize lack of knowledge about male infertility |  |
| More aware to perceive the needs for psychological support |  |
| Offered instruments for the psychological evaluation of patients | **Better screening/evaluation/diagnosis** |
| It has helped to identify clients who have such problems |  |
| We question the risk factors more systematically |  |
| Screen psychological symptoms with appropriate instruments |  |
| Screening for social and lifestyle before treatment more rigorous |  |
| We ask more how patients emotionally feel |  |
| Allowed to detect couples potentially at risk of giving up treatment, and thus improved the potential for adherence to treatment |  |
| We use the FertiQol |  |
| Offer more psychological support to patients having fertility treatment | **Better psychosocial support/counselling** |
| Counsel patients |  |
| Proper counseling |  |
| Made counselling more available |  |
| Taking good care for patients with infertility had improved outcome and treatment results |  |
| We look for the patient to feel good |  |
| Focus on the psychosocial aspect more |  |
| Psychosocial counselling done throughout treatment |  |
| Better care |  |
| The emotional life of patient |  |
| Pay attention |  |
| How to handle those who do not get positive results |  |
| Better options for unsuccessful treatments |  |
| How to deal with failure |  |
| improve my methods of assistance on higher quality level |  |
| Personalised counselling on treatment plans and results with clinic staff |  |
| More referrals for psychological support | **Better patient-centred care** |
| Refer patients at risk of emotional problems to specialised psychosocial care |  |
| Proper indication |  |
| I am aware of couple's psychological health to refer them to specialised psychologist |  |
| Cater to their psychosocial needs |  |
| We prioritize your preferences |  |
| Involve both partners in the treatment process |  |
| Providing written information | **Better education/information provision** |
| I spend more time for patient's briefing |  |
| Recall the best way of speaking with couples | **Better communication** |
| Spending more time for listening to patients |  |
| It has improve communication |  |
| It was an improvement of the relation with the patient |  |
| To represent patients psychosocial interests in medical team | **More interdisciplinary/team work** |
| We had several meetings with the psychologist |  |
| Allowed in a simple way to clarify about the psychosocial universe of infertility to the work team |  |
| I could transmit it to the team, and gave to everybody the summary |  |
| Institutional rapports |  |
| Counsel the staff in the clinic |  |
| Patients survey on counselling provision | **Clinical auditing** |
| New counselling room | **New infrastructures** |
| Health organization | **Non codable** |
| Clear objectives |  |
| Recommendations written in the guideline give to clinicist an instrument to make patients with infertility psychologically stable. |  |
| To do in real time |  |
| Predictability |  |
| Channel their difficulties |  |
| Emotions about pregnancy |  |
| So it can be a document we can give in reference to improve the way we ar taking care of the patients |  |
| Better results in infertility managements |  |
| Scientific approach |  |
| Access |  |

| **Supplementary Table SV**  Barriers to implement the Routine Psychosocial Care guideline reported by participants. | |
| --- | --- |
| **Participants’ answers** | **Category** |
| **Guidelines** |  |
| Some unclear topics | **Unclear/difficult to understand** |
| Not well clear |  |
| Not attractive to read: difficult and unclear layout at first sight |  |
| It is to understand |  |
| Same as before (It is very big) | **Too long** |
| Too large, needs short version of recommendation |  |
| To large |  |
| Limited evidence | **Lack of evidence** |
| Limited evidence on the topic |  |
| Limited solid evidence; a great deal of good research that did not make it to the strict guidelines criteria |  |
| Not enough evidence to make strong direct and practical recommendations to clinical staff - moved from clinic |  |
| Not enough evidence to make strong direct and practical recommendations to clinical staff - moved from staff |  |
| Language | **Language/translation** |
| The pocket guideline is useful but It has been translated in French on an artisanal way |  |
| Language |  |
| Not everybody knows English - moved from staff |  |
| Language - overly monitoring - moved from patient |  |
| We didn’t translate it for the patients (not enough time...,) - moved from patient |  |
| Only a small amount of my clients are fluent in English enough to appreciate the guidelines. A translation in Bulgarian would be useful. - moved from patient |  |
| Language - moved from patient |  |
| Not all our patients know English - moved from patient |  |
| Language limitation - moved from patient |  |
| Guideline topic is not as interesting as other issues although it is very important | **Topic not relevant/inappropriate/not priority** |
| Aware of the content and it largely matches the Australian guidelines for many topics. - moved from staff |  |
| Not relevant - moved from staff |  |
| **Clinical setting & system** |  |
| Cost effectiveness | **Costs/financial constrains** |
| Costs |  |
| Change of some routine without financial benefit to the clinic |  |
| Costs |  |
| It cost money to produce it, no energy to fight for it |  |
| Costs |  |
| No specific equipment for this topic | **Lack of infrastructure/equipment** |
| More personnel with special training is needed to provide adequate consultation to the couple. | **Lack of trained/specialised staff** |
| Own routine methods | **Culture/norms** |
| Within a general hospital follow general rules |  |
| Against local habits |  |
| Private clinic with in house therapist |  |
| The clinics that I know don't use the guidelines - moved from staff |  |
| No one to reinforce the use of the guidelines | **No champion/responsible person** |
| They don’t really apply to our set of Indian /Asian patients - moved from staff | **Not relevant to clinic patient population** |
| **Staff** |  |
| Lack of expertise | **Lack of knowledge/expertise** |
| Experience |  |
| Lack of knowledge |  |
| Lack of knowledge |  |
| Lack of knowledge |  |
| Lack of time | **Lack of time** |
| No real time to do the promotion more than what we did (translation, and artisanal pocket book, presented to the team..) |  |
| Need extra time with the patient - moved from clinic |  |
| Limited time - moved from clinic |  |
| Not enough time to explain - moved from clinic |  |
| Not enough time for the midwives to question the emotions of patients - moved from clinic |  |
| Time to read the guidelines, establish a working group to implement the guideline - moved from clinic |  |
| Lack of power | **Lack of power** |
| Or is eager to try something new | **Lack of interest/motivation** |
| Some personal not interested |  |
| Difficulty on engage medical team with |  |
| **Patient** |  |
| Find it difficult to co relate due to ethical differences | **Culture/norms** |
| Local habits |  |
| Against local habits |  |
| Culture |  |
| Against local habits |  |
| Cultural difference between nations |  |
| Couples enjoy the Guidelines, but not all couples have instruction to understand them. In this case I explain better and I do not give them the Guidelines |  |
| Cultural - moved from staff |  |
| This increases the final cost for the couple | **Costs** |
| Cost of some procedures for patients |  |
| Cost |  |
| Patients are often refractory to psychological support | **No interest/resistance** |
| Not cooperative |  |
| Patients can be impatient to read especially for patients who are infertile long term |  |
| Difficult for the patients to understand that IVF is a psychologically stressful procedure-they avoid talking about that issue |  |
| Resistent |  |
| **Non codable** |  |
| Useful | **Non codable** |
| Help patients |  |
| We do use the recommendations. |  |
| We do use the guidelines. |  |
| I do not work on a Clinic |  |
| The clinicians may not be awarded for this topic so guidelines may create awareness for clinicians |  |
| Clinican |  |
| Lack of clinician |  |
| Couples enjoy the Guidelines |  |
| We tend to suggest Australian resources. It would be fabulous to have man international online support group, blog or forum that patients could rely on as a valid source of information. |  |

| **Supplementary Table SVI**  Support to implement the Routine Psychosocial Care guideline reported by participants. | |
| --- | --- |
| **Participants’ answers** | **Category** |
| **Guideline** |  |
| Guide where to look for information | **Clearer and concise information** |
| More concise guideline |  |
| Clear |  |
| Same as before (Small flowchart to laminate in clinic) |  |
| Short crisp recommendation for quick reference |  |
| More clear overview, different layout that makes it more attractive and less confusing |  |
| More concise written summary - moved from staff |  |
| Step by step - moved from staff |  |
| Spread | **Better dissemination of the guidelines** |
| Social media, Facebook, emails... |  |
| I would like it to contain more information | **Additional information** |
| A better leaflet - moved from patient | **Patient version/leaflets/information** |
| Leaflets - moved from patient |  |
| Handout to patients telling about the facts or videos which can be given to them as a CD – moved from patient |  |
| Leaflets for patients - moved from guidelines |  |
| An illustrated guide for patient - moved from clinic |  |
| App, where we can look up quickly - moved from staff | **App/other digital forms** |
| Translation to different languages | **Translation/language** |
| A translation |  |
| Translation |  |
| The pocket one, in French, |  |
| Same (The pocket one, in French) - moved from clinic |  |
| Same (The pocket one, in French) - moved from staff |  |
| Translation to different languages - moved from staff |  |
| Multiple language translation with content validation - moved from patient |  |
| Translation to different languages - moved from patient |  |
| Same for the patient (The pocket one, in French) - moved from patient |  |
| **Clinic** |  |
| Standardised clinical. good recommendations of clinics it has to be supported or certified | **Endorsement of guidelines by local authority** |
| Implementation of the guide by local authorities |  |
| ESHRE recommendation paper for clinic |  |
| Affiliated psychologists. | **Specialized staff** |
| Team working, getting buy in from the team makes a huge difference, the patient information helps all staff to understand more about the needs of our patients, from receptionist to accounts - much easier reading for non-clinical staff | **Multidisciplinary/team work** |
| **Staff** |  |
| More time for consultation – moved from clinic | **Time** |
| Enough time for each patient – moved from clinic |  |
| Time, cost (in time) |  |
| Group teaching of all staff - moved from clinic | **Education/courses** |
| Knowledge, courses moved from clinic |  |
| E-learning course - moved from clinic |  |
| Lack of knowledge |  |
| Have knowledge |  |
| E-learning course |  |
| Encouragement | **Motivational support** |
| Interest |  |
| **Patient** |  |
| To better understand their disease | **Education/awareness** |
| Health education |  |
| Cooperative | **Attitude/cooperation** |
| **Support not needed** |  |
| Not required. | **Support not needed** |
| Not required. |  |
| Not required. |  |
| **Non codable** |  |
| Benefit | **Non codable** |
| More space for psychosocial aspects annual meetings - not as a separate session, but included in the main sessions! |  |
| Do not need specific equipment |  |
| Treatment options |  |
| Clinic |  |
| Global guidelines |  |
| With the use of these guidelines clinicians or who uses medical staff also can feel more confident |  |
| Not relevant |  |
| Some patients want to talk or communicate much more they convince that they will be covered for all issues about infertility |  |
| Usage |  |
| Against habits |  |
| Support style patient friendly "hub" |  |

| **Supplementary Table SVII**  Changes in practice due to applying the Premature Ovarian Insufficiency guideline reported by participants. | |
| --- | --- |
| Participants’ answers | Category |
| The nomenclature | **Increased awareness/knowledge** |
| Knowledge |  |
| I was looking for literature and textbook for POI but this guideline is faster and easier to reach from just ESHRE website so time |  |
| Well defined POI |  |
| Evidence to support care |  |
| Awareness |  |
| Right prescriptions to find out a potential cause | **Better screening/evaluation/diagnosis** |
| Diagnostic thresholds are more realistic |  |
| Diagnosis |  |
| Clarity on diagnosis of POF |  |
| Better investigation |  |
| Ruling out all possible causes of POF |  |
| I added some lab test of ethology autoimmune POI |  |
| Genetic testing you need to ask |  |
| Clear and faster diagnosis |  |
| Step by step evaluation of POF |  |
| Better diagnosis of ovarian failure |  |
| Preventive measures for estrojen depletion | **Better general treatment** |
| POI and ovarian stimulations |  |
| Karyotyping for all POI patients |  |
| Management steps |  |
| Added karyotyping to those in who the cause was not iatrogenic |  |
| Treatment duration |  |
| Treatment |  |
| Offering hormone terapi |  |
| Management of POI |  |
| Greater Clarity on HRT in POI |  |
| Treatment of POI especially systematic cardiovascular and other medical issues also sexuality well being for clinical practice |  |
| Referring them to specialist fetomaternal units as high risk pregnancies |  |
| Proper treatment |  |
| Medicine dosage |  |
| HRT for all POI |  |
| More referring to IVF |  |
| Counseling Psychosocial | **Better psychosocial support/counselling** |
| Better patient counseling |  |
| Began counselling of relatives on possible implications for them |  |
| Counselling to address concerns among close family members |  |
| Shorter time for decision making | **Better patient-centred care** |
| Management of patients |  |
| Decision making |  |
| Follow up of women with POI |  |
| Providing information | **Better education/information provision** |
| Better patient education |  |
| Better explanations for patients |  |
| Up to date informations to the patient |  |
| Education |  |
| Selection | **Non codable** |
| Advocate |  |
| Evidence based studies |  |
| Suggesting conducts |  |

| **Supplementary Table SVIII**  Barriers to implement the Premature Ovarian Insufficiency guideline reported by participants. | |
| --- | --- |
| **Participants’ answers** | **Category** |
| **Guidelines** |  |
| Tests for accompanying immunological conditions are not clear - moved from clinic | **Unclear/difficult to understand** |
| Lack of understanding - moved from patient |  |
| Be able to understand - moved from patient |  |
| Difficult to understand - moved from patient |  |
| Very long | **Too long** |
| Heavy to read |  |
| As before (It is very big) |  |
| Not conclusive | **Lack of evidence** |
| Limited evidence on topic |  |
| Evidences |  |
| We have our own patient resources, in local languages. | **Language/translation** |
| The language barrier is a bit difficult - moved from patient |  |
| Not translated into French - moved from patient |  |
| Not in German - moved from patient |  |
| Language... only English - moved from patient |  |
| Language barrier - moved from patient |  |
| Language - moved from patient |  |
| Language - moved from patient |  |
| Irrelevant - moved from staff | **Topic not relevant/inappropriate/not priority** |
| I believe patients would find it useful to have a document to share with their GP around addition support, onward referral, future health management relating to a POI diagnosis - moved from patient | **Lack of patient friendly version/materials** |
| Awareness of guidelines must be spread through clinicians. | **No dissemination/awareness** |
| Again some clinics can not be aware of ESHRE guidelines. - moved from clinic |  |
| Patient is info of guidelines must be spread through social media they can get this info from just clinicians - moved from patient |  |
| **Clinical setting & system** |  |
| Costs | **Costs/financial constrains** |
| Cost factor of the tests; fragile X and |  |
| Cost |  |
| Cost |  |
| **Staff** |  |
| Lack of knowledge | **Lack of knowledge/expertise** |
| Lack of knowledge |  |
| Lack of knowledge |  |
| Lack of expertise |  |
| time | **Lack of time** |
| Time |  |
| No task for a midwife, task for MD | **Not personally applicable/relevant** |
| As embryologist I do not speak to patients about these topics |  |
| **Patient** |  |
| Patients think that having premature menopause is a taboo in india; they still do not come to the right person for advice | **Culture/norms** |
| High cost of genetic testing | **Costs** |
| Costs |  |
| **No barriers** |  |
| None | **No barriers** |
| None |  |
| None |  |
| No |  |
| No |  |
| I do not see barriers |  |
| None |  |
| No |  |
| No |  |
| I do not see barriers |  |
| Nothing |  |
| None |  |
| None |  |
| No |  |
| No |  |
| I do not see barriers |  |
| None that I am aware of |  |
| No |  |
| **Non codable** |  |
| See answer 19 | **Non codable** |
| Fragile x mutation |  |
| See answer 19 |  |
| Will do so, hadn't as an oversight |  |
| See answer 19 |  |
| See answer 19 |  |
| Information |  |

| **Supplementary Table SIX**  Support to implement the Premature Ovarian Insufficiency guideline reported by participants. | |
| --- | --- |
| **Participants’ answers** | **Category** |
| **Guidelines** |  |
| What tests are basic and minimal for a country like India where majority of the issues are cost driven | **Clearer and concise information** |
| Step by step of how to implement the guidelines |  |
| As before (Small flowchart to laminate in clinic) |  |
| A quick summary - moved from staff |  |
| Show iz – moved from clinic | **Better dissemination of the guidelines** |
| Guidelines must be spread through social media especially to IVF fertility centres – moved from clinic |  |
| Should provide or suggest a number of treatment regimens for the infertility issues in POI patients | **Additional information** |
| Need more elaboration. Kindly review and u will know. |  |
| Newspapers or social media especially Facebook - moved from patient | **Patient version/leaflets/information** |
| More patient friendly reading material - moved from patient |  |
| Information brochure - moved from patient |  |
| App - moved from patient |  |
| A leaflet - moved from patient |  |
| Podcast or small video guidance for patients - moved from clinic |  |
| Digital | **App/other digital formats** |
| Digital |  |
| App - moved from staff |  |
| Several languages - moved from patient | **Translation/language** |
| Patient version in Russian - moved from patient |  |
| Need translation in our language - moved from patient |  |
| Issue in German - moved from patient |  |
| A translated version - moved from patient |  |
| Clinical experience part can be added (not all evidence based) | **Recommendations based on expert opinion** |
| Updating and revising these guidelines | **Updating** |
| Specific diagnostic criteria using emerging new technologies or biomarkers |  |
| **Clinic** |  |
| More insurance covered tests | **Funding** |
| **Staff** |  |
| Learn and take a time for it | **Time** |
| **Patient** |  |
| Understand it | **Education/awareness** |
| **Support not needed** |  |
| The way it is | **Support not needed** |
| None |  |
| No |  |
| The way it is |  |
| No |  |
| The way it is |  |
| None |  |
| No |  |
| **Non codable** |  |
| See answer 19 | **Non codable** |
| Read it |  |
| See answer 19 |  |
| Costs |  |
| Clinical guidelines |  |
| To give confidence that medical staff will do wrong and cover all aspects of this disease and they will all convince patients |  |
| See answer 19 |  |
| Not relevant |  |
| Irrelevant |  |
| See answer 19 |  |

| **Supplementary Table SX**  Dissemination, implementation and impact of the four published ESHRE guidelines, by clinicians (N = 260). | | | | | |
| --- | --- | --- | --- | --- | --- |
|  |  | ENDO | RPC | POI | RPL |
| Know was published | q/n^a^ | 240/260 | 66/122 | 75/104 | 77/95 |
|  | % [95% CI] | 92.31 [88.42 - 94.97] | 54.10 [45.27 - 62.68] | 72.12 [62.83 - 79.83] | 81.05 [72.03 - 87.67] |
| Downloaded | q/n^b^ | 214/240 | 42/66 | 59/75 | 58/77 |
|  | % [95% CI] | 89.17 [84.60 - 92.50] | 63.64 [51.58 - 74.19] | 78.67 [68.12 - 86.42] | 75.32 [64.65 - 83.60] |
| Use in daily practice | q/n^b^ | 173/240 | 30/66 | 51/75 | - |
|  | % [95% CI] | 72.08 [66.09 - 77.38] | 45.45 [34.02 - 57.38] | 68.00 [56.79 - 77.46] | - |
| Changes in practice | q/n^b^ | 133/240 | 24/66 | 32/75 | 40/77 |
|  | % [95% CI] | 55.42 [49.09 - 61.57] | 36.36 [25.81 - 48.42] | 42.67 [32.10 - 53.95] | 51.95 [40.96 - 62.75] |
| Perceived patient benefit | q/n^b^ | 87/240 | 21/66 | 27/75 | - |
|  | % [95% CI] | 36.25 [30.43 - 42.50] | 31.82 [21.85 - 43.79] | 36.00 [26.06 - 47.30] | - |
|  | *M* | 4.32 | 4.38 | 4.41 | - |
|  | *SD* | 0.67 | 0.74 | 0.80 | - |
| Referred to patients | q/n^b^ | 28/240 | 12/66 | 21/75 | - |
|  | % [95% CI] | 11.67 [8.20 - 16.34] | 18.18 [10.72 - 29.15] | 28.00 [19.10 - 39.04] | - |
| *Note.*  q = number of participants replying yes to question.  ^a^ n = number of valid answers (i.e., not missing): ENDO = 260, RPC = 122, POI = 104, RPL = 95.  ^b^ n = number of participants who reported knowing the guidelines were published: ENDO = 240, RPC = 66, POI = 75, RPL = 77.  ENDO = Endometriosis, RPC = Routine Psychosocial Care, POI = Premature Ovarian Insufficiency, RPL = Recurrent Pregnancy Loss. | | | | | |
